# Continent vesicovaginal fistula

**DOI:** 10.1590/S1679-45082013000100022

**Published:** 2013

**Authors:** Luís Gustavo Morato de Toledo, Victor Espinheira Santos, Paulo Eduardo Gourlat Maron, Bruno César Vedovato, Moacyr Fucs, Marjo Deninson Cardenuto Perez

**Affiliations:** 1 Faculdade de Ciências Médicas da Santa Casa de São Paulo, São Paulo, SP, Brazil; Hospital Ipiranga, São Paulo, SP, Brazil; Hospital Ipiranga, São Paulo, SP, Brazil; 2 Santa Casa de Misericórdia de São Paulo, São Paulo, SP, Brazil

**Keywords:** Vesicovaginal fistula, Urinary incontinence, Case reports

## Abstract

Vesicovaginal fistula is an abnormal communication between the bladder and vagina and represents the most frequent type of fistula in the urinary tract. The most common cause in Brazil is iatrogenic fistula, secondary to histerectomia. Classically these women present continuous urinary leakage from the vagina and absence of micturition, with strong negative impact on their quality of life. We present a case of totally continent vesicovaginal fistula, with a follow-up of 11 years with no complications.

## RESUMO

A fístula vesicovaginal é uma comunicação anormal entre a bexiga e a vagina, sendo o tipo mais comum de fístula do trato urinário. A causa mais frequente no Brasil é a iatrogênica, secundária à histerectomia. Classicamente, as mulheres nessa condição, apresentam perda urinária contínua pela vagina e ausência de micção, com forte impacto negativo na qualidade de vida. Apresentamos um caso de fístula vesicovaginal totalmente continente, com seguimento de 11 anos, sem complicações.

## INTRODUCTION

Vesicovaginal fistula (VVF) is the most common type of urinary tract fistula. It can result from obstetric trauma, surgery, infection, malignancy or congenital anomalies^(1)^. In developed countries, this condition most commonly results from gynecological surgery, particularly hysterectomy. By contrast, in developing nations, VVF is associated with obstetric complications, such as prolonged labor^(1,2)^. There are at least 3 million patients with VVF in developing countries, with approximately 33 thousand new cases reported annually in Sub-Saharan Africa alone^(1)^.

Women with vesicovaginal fistula present a continuous leakage of urine from the vagina, usually with absence of urethral voiding, resulting in devastating consequences with respect to their physical and psychological health^(1)^. The classical treatment is surgery and the vaginal route is currently the preferred option^(3)^. Despite a thorough literature review, the authors were unable to find any reports of VVF patients who were, at the same time, fully continent.

The present study is a case report of a 62-year-old patient who, after undergoing multiple abdominal and gynecological surgeries, experienced a vesicovaginal fistula in the absence of urinary incontinence.

## CASE REPORT

A 62-year-old female patient from São Paulo (SP), Brazil, had a previous history of hysterectomy for fibroids, with six abdominal interventions undertaken due to surgical complications, and the final procedure was performed in February 2000. Two weeks following her hysterectomy, she presented vaginal urine leakage with an absence of urethral voiding for 4 months, after which she began to notice a progressive and spontaneous improvement until full continence was eventually achieved.

**Figure 1:**
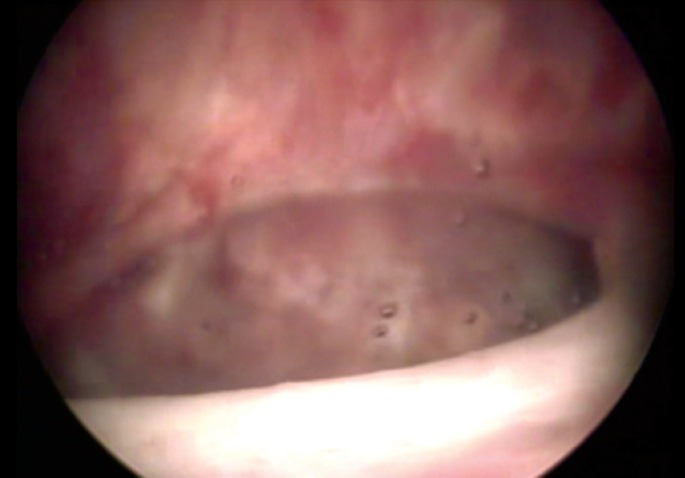
Vesicovaginal fistula 4-cm in diameter

**Figure 2:**
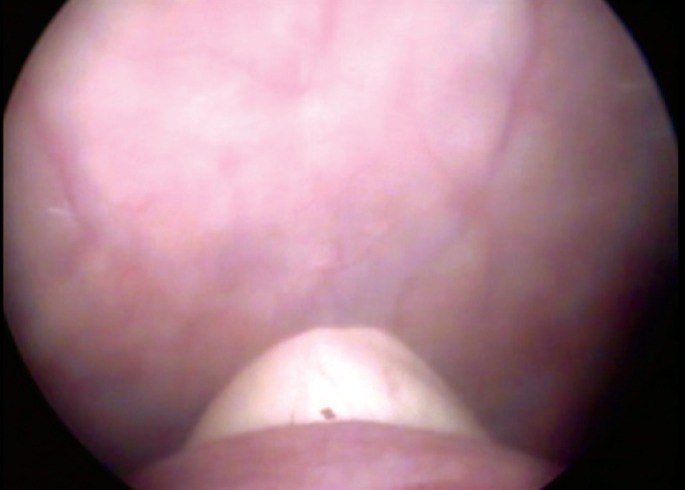
Index finger inserted in the vagina through the fistula

**Figure 3:**
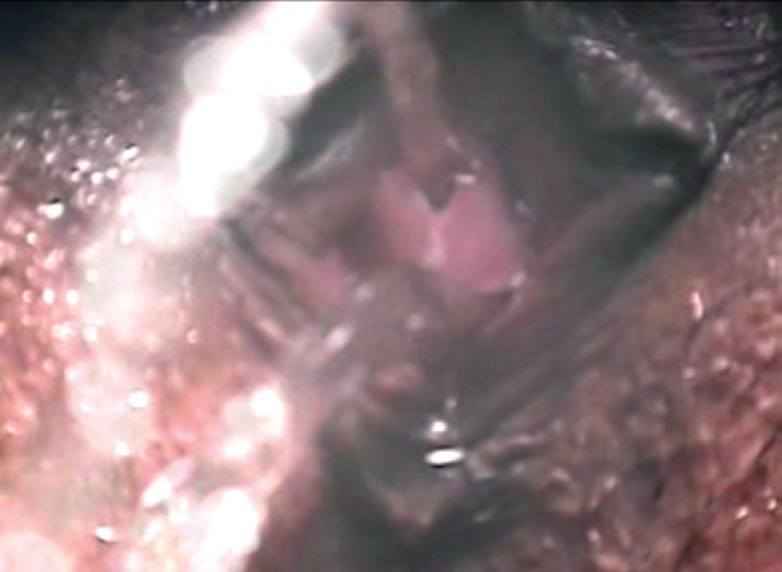
Voluntary micturition via both the vagina and urethra

**Figure 4:**
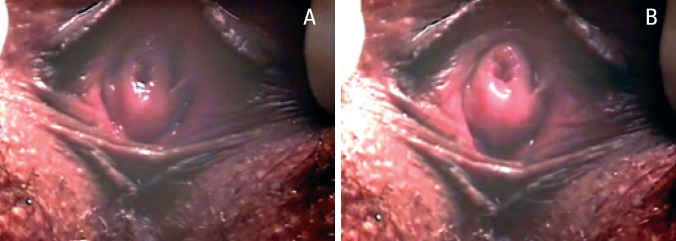
Absence of urinary leakage with a full bladder: a) rest and b) cough

Cystoscopy revealed a single fistula, 4cm in diameter, located above the trigone. The urethral meatus was undisturbed and correctly positioned. She did not have any urinary leakage during a full bladder stress maneuver and, upon demand, urination occurred simultaneously when requested by both vaginal and urethral routes (Figures 1 to 4).

The patient declined surgical treatment due to being fully continent and she was not afraid of complications. She experienced profound aversion to the operating room ever since undergoing six abdominal surgeries for complications arising from her hysterectomy.

The patient's upper urinary tract was assessed and found to be well-preserved both in terms of function and anatomy.

The patient was followed up clinically and has undergone annual cystoscopic evaluations over the last 11 years, which revealed no signs of malignant degeneration of the bladder urothelium or any lower urinary tract symptoms. Her urine culture was positive for *Escherichia coli*, yet antibiotic treatment has been unwarranted to date.

The patient reported a satisfactory quality of life, being fully continent even during sexual intercourse. Furthermore, even under exertion, she did not need absorbent pads and was able to voluntarily urinate simultaneously through vaginal and urethral voiding (Figures 3 and 4).

## DISCUSSION

The present case is not only highly unusual and intriguing, but also, to the best of our knowledge, unprecedented. Deficiencies in public healthcare system in Brazil are the underlying reason for leading to this case, and had this patient come to our department due to incontinence, she would likely have agreed to undergo surgical treatment. When first seen at the Urology Department of the hospital Santa Casa de Misericórdia de São Paulo, 6 months after her last surgical intervention, she was already fully continent and had considered herself cured.

There are some therapeutical options for VVF, such as bladder catheterization in cases of small fistulas, and surgical repair by abdominal, vaginal or combined approach^(3)^. Simple observation was not considered a valid therapeutic choice. Our understanding was that her continence would be transient and the patient was informed of the risk of urinary tract infection, impaired renal function and possible malignant degeneration of the urothelium. However, no argument was able to persuade the patient; the phobia of surgery caused her to reject any type of invasive treatment. In fact, she even refused to enter the operating theater for routine testing, such as cystoscopy and cystourethrography.

This patient exhibits unusual and extraordinary voluntary control and strength of her perineal muscles, especially the deep transverse perineal muscle and pubovisceral bundle of the levator ani muscle. This ability was probably developed spontaneously, since this quality of muscle training is extremely difficult to achieve in patients with stress urinary incontinence (SUI), even with the aid of physical therapy by qualified professionals. However, rehabilitation of the pelvic floor muscles, through strength training programs, is considered the first choice of treatment of women with SUI ^(4,5)^.

Injuries to the neurological and fascial components of the pelvic floor, in addition to muscle damage, may explain the difficult responding to physical therapy in patients with SUI^(6)^. Thus, it would be reasonable to prophylactically encourage perineal exercises^(7)^ when the anatomical structures are intact and not after injury, as is usually the case.

Full continence, even during sexual intercourse, was an impressive finding in this patient, which was repeatedly questioned. The patient, along with her husband, reported no incontinence during intercourse with a weekly frequency of sexual activity. The voiding of the bladder and vagina shortly before intercourse does not justify her continence, due to the sealing of the vaginal opening surrounding the penis, which is secondary to the action of the pelvic muscles as mentioned above.

Due to the known consequence of urinary incontinence and its subsequent repercussions as a result of VVF, which did not occur in this patient, we plan to continue to follow this patient up indefinitely. Although chronic bladder colonization may be comparable to asymptomatic bacteriuria, there is no precedent for potential urothelial malignancy, which remains a concern.

## CONCLUSION

To our knowledge, this is the first case of vesicovaginal fistula reported in a fully continent patient. Chronic communication between bladder and vagina did not imply any malignant or infectious complications in either organ.
